# Extracellular Matrix Biomarker, Fibulin-1, Is Closely Related to NT-proBNP and Soluble Urokinase Plasminogen Activator Receptor in Patients with Aortic Valve Stenosis (The SEAS Study)

**DOI:** 10.1371/journal.pone.0101522

**Published:** 2014-07-11

**Authors:** Ruan Kruger, Lars M. Rasmussen, William S. Argraves, Jesper Eugen-Olsen, Olav W. Nielsen, Adam Blyme, Ronnie Willenheimer, Kristian Wachtell, Michael H. Olsen

**Affiliations:** 1 Hypertension in Africa Research Team (HART), North-West University, Potchefstroom, South Africa; 2 The Cardiovascular and Metabolic Preventive Clinic, Department of Endocrinology, Centre for Individualized Medicine in Arterial Diseases (CIMA), Odense University Hospital, Odense, Denmark; 3 Department of Clinical Biochemistry and Pharmacology, Centre for Individualized Medicine in Arterial Diseases (CIMA), Odense University Hospital, Odense, Denmark; 4 Medical University of South Carolina, Department of Cell Biology, Charleston, South Carolina, United States of America; 5 Clinical Research Centre 136, Hvidovre Hospital, Copenhagen, Denmark; 6 Department of Cardiology, Bispebjerg Hospital, Copenhagen, Denmark; 7 Department of Cardiology, Glostrup Hospital, Copenhagen, Denmark; 8 Lund University and Heart Health Group, Malmö, Sweden; 9 Department of Medicine, Glostrup Hospital, Copenhagen, Denmark; Sapienza University of Rome, Italy

## Abstract

**Background:**

Fibulin-1, a circulating extracellular matrix glycoprotein, has been associated with arterial disease and elevated N-terminal prohormone B-type natriuretic peptide (NT-proBNP) in diabetes. Soluble urokinase plasminogen activator receptor (suPAR), a marker of inflammation, has been associated with subclinical atherosclerosis. Therefore, we aimed to explore the interplay between these biomarkers and mild to moderate aortic valve stenosis (AS).

**Methods:**

In 374 patients with mild to moderate AS, we investigated the relationship of fibulin-1 with NT-proBNP, levels of suPAR and the degree of AS at baseline and after one and four years of treatment with Simvastatin 40 mg and Ezetimibe 10 mg or placebo.

**Results:**

During treatment, fibulin-1 became more closely associated with NT-proBNP (β_year0_ = 0.10, p = 0.08, β_year1_ = 0.16, p = 0.005, β_year4_ = 0.22, p<0.001) and suPAR (β_year0_ = 0.05, p = 0.34, β_year1_ = 0.16, p = 0.006, β_year4_ = 0.13, p = 0.03) at the expense of the association to aortic valve area index (AVAI) (β_year0_ = −0.14, p = 0.005, β_year1_ = −0.08, p = 0.11, β_year4_ = −0.06, p = 0.22) independently of age, gender, creatinine, and serum aspartate aminotransferase (Adj.R_year0_^2^ = 0.19, Adj.R_year1_^2^ = 0.22, Adj.R_year4_^2^ = 0.27). Fibulin-1 was unrelated to aortic regurgitation, left ventricular mass, and ejection fraction. In patients with baseline AVAI<0.58 cm^2^/m^2^ (median value), fibulin-1 was more closely associated to NT-proBNP (β_year0_ = 0.25, β_year1_ = 0.21, β_year4_ = 0.22, all p<0.01), and suPAR (β_year0_ = 0.09, p = 0.26, β_year1_ = 0.23, β_year4_ = 0.21, both p<0.01) independently of age, gender, AST and treatment allocation.

**Conclusions:**

Increased levels of fibulin-1 were independently associated with higher levels of suPAR and NT-proBNP especially in patients with lower AVAI, suggesting that fibulin-1 may be an early marker of AS as well as cardiac fibrosis secondarily to elevated left ventricular hemodynamic load.

## Introduction

Aortic stenosis is a slow progressing condition developing with increasing age and abnormal calcium build up in the aortic valve [1], [2]. Consequences of aortic stenosis normally include myocardial hypertrophy and fibrosis [3], [4], finally cascading into heart failure, angina and sudden death [5]. Particularly soluble urokinase plasminogen activator receptor (suPAR)) has an overlapping interaction with atherosclerotic formation [6] and stiffness related to aortic stenosis [3], [4], [7]. Adverse hemodynamic alterations contribute to alter the integrity of structural components necessary to maintain healthy cardiovascular function, hence promoting extracellular matrix remodelling [8].

Fibulin-1, a calcium-binding extracellular matrix glycoprotein, recently received interest on its association with elastin fibres within cardiovascular tissue and fibrinogen cross-linking [9], [10]. A study on rats found that levels of fibulin-1 are down-regulated in events related to cardiac ischemia [8]. However, no data is available on its potential involvement in fibrogenesis of heart failure progression. In addition, fibulin-1 is related to the amino-terminal prohormone B-type natriuretic peptide (NT-proBNP) in type 2 diabetes [11] and African patients subjected to early vascular ageing [12]. In cardiovascular disease states subjected to extracellular matrix turnover, fibulin-1 participates in an augmenting fashion and gradually contributes to the progression of tissue remodelling.

Due to the known associations of fibulin-1 with cardiac overload, as well as restrictive cardiomyopathy and decreased left ventricular (LV) systolic function in patients with symptomatic severe aortic stenosis [13], we hypothesize that fibulin-1 could be a potential biomarker of either early cardiac or valvular fibrosis. Currently, aortic stenosis is quantified by echocardiography demanding trained technicians or physicians. Therefore, biomarkers to screen for disease onset and progression of aortic stenosis would be very helpful especially in hospitals and clinics with limited health care facilities. Currently clinicians have a dilemma because many people slowly develop asymptomatic aortic valve sclerosis with increasing age, but may not necessarily progress to clinical pathology, whereas in some patients the process starts earlier and/or progress faster. Furthermore, the relationships between echocardiography findings and symptoms as well as the development of concomitant LV hypertrophy and improvement of symptoms vary between individuals. Therefore, new tools such as new biomarkers are needed to predict both anatomical and symptomatic progression as well as the cardiovascular risk in order to time operation better.

The aim of this study was to investigate whether fibulin-1 was associated with NT-proBNP, suPAR and echocardiography measures in patients with mild to moderate aortic valve stenosis before and during lipid-lowering treatment.

## Methods

### Study design and patient population

*Ethics statement* – All patients gave written informed consent and the ethical committees from all participating countries (Norway, Sweden, Denmark, Finland, Germany, the United Kingdom, and Ireland) approved the study.

The current study formed part of the Simvastatin and Ezetimibe in Aortic Stenosis (SEAS) trial which was originally designed to investigate the potential of lipid lowering drug treatment to minimise aortic valve replacements and to curb the risk of cardiovascular disease and death. The outline of the larger study and SEAS protocol was published in detail elsewhere [18]. Participants aged 45 years and older were included in this study, whereas patients with known rheumatic disease or congenital cardiomyopathy were excluded. Patients aged between 45–85 years, with asymptomatic aortic stenosis, as determined with Doppler ultrasound (aortic peak velocity ≥2.5 and ≤4.0 m/sec), were included. A selection of 396 patients (with the relevant measured biochemical data for this study) was included after pairing groups with mild versus moderate aortic stenosis by age, gender and treatment allocation. After excluding those with missing data a total of 374 patients remained in the current analysis.

### Echocardiography and hemodynamic measurements

The standardized echocardiography protocol of the SEAS study was previously published [14], [15]. All images were recorded and sent for central, blinded interpretation by qualified technicians at the SEAS echocardiography core laboratory at the Haukeland University Hospital in Bergen, Norway. Captured data was analysed using off-line digital workstations with Image Arena (TomTec Imaging Systems, Unterschleissheim, Germany) software. The American Society of Echocardiography guidelines were used to quantify LV internal dimensions as well as posterior and septal wall thickness along with LV volumes and ejection fraction (determined with the Simpson's method) [16]. LV mass was calculated by using the end-diastolic LV dimensions with an anatomically validated formula [17], [18]. LV hypertrophy was determined by calculating the LV mass index for men (≥116 g/m^2^) and women (≥104 g/m^2^) by dividing LV mass by body surface area [19]. Aortic valve area index (AVAI) was calculated by determining the valve area using the continuity equation with the time velocity/time integral ratio [20] and then indexed for body surface area. Aortic regurgitation was assessed by colour Doppler using 4-point grading systems, previously described by Jones et al. [21]. After echocardiographic examination, clinical blood pressures were measured in supine position.

### Biochemical analyses

For this study we determined serum creatinine, serum aspartate aminotransferase and serum high-sensitivity C-reactive protein (CRP) levels with the Konelab autoanalyzer (Thermo Fisher Scientific Oy, Vantaa, Finland). The estimated glomerular filtration rate was determined with the Modification of Diet in Renal Disease (MDRD) Study equation: eGFR  = 175× (Standardized S_cr_)^-1.154^× (age)^−0.203^× (0.742 if female). EDTA-coagulated plasma was used to determine fibulin-1 levels by using a sandwich immunoassay [11]. The Elecsys proBNP sandwich immunoassay was used on an Elecsys 2010 (Roche Diagnostics, Mannheim, Germany) to determine the serum NT-proBNP concentration of each participant. Plasma (EDTA) suPAR levels were measured using the suPARnostic ELISA kit (ViroGates, Copenhagen, Denmark).

### Statistical analysis

All statistical analyses were performed with the IBM SPSS Statistics, Version 22 (IBM Corporation, Armonk, New York). Non-Gaussian variables (tested by the method of skewness (normal range: −0.8 to 0.8)) were fibulin-1 (0.893), NT-proBNP (2.97), suPAR (1.19), AVAI (borderline, 0.79) and LV mass index (1.07). These variables were logarithmically transformed prior to any statistical analysis. Associations of fibulin-1 with markers of cardiac overload and inflammation were tested for interaction with severity of aortic stenosis, gender and treatment allocation by performing the appropriate interaction terms. Χ^2^ tests were used to compare proportions of dichotomous or categorical variables and independent T-tests to compare the means of continuous variables between groups. An analysis of covariance (ANCOVA) analysis was used to determine fibulin-1′s relationship with NT-proBNP and suPAR independent of baseline variables. Pearson correlations between fibulin-1, NT-proBNP, suPAR and echocardiography data were plotted in scatterplot graphs and z-scores were tested. Multiple regression analyses were performed to determine independent relationships between fibulin-1 and main independents of interest (NT-proBNP and suPAR) with the relevant covariates for each model.

## Results

The basic clinical characteristics of the study sample are listed in Table 1. The total group was divided according to the predetermined median (0.58 cm^2^/m^2^) of the AVAI and categorized in a group with mild (≥0.58 cm^2^/m^2^) versus a group with moderate (<0.58 cm^2^/m^2^) aortic stenosis. Hemodynamic measures (systolic and diastolic blood pressure) were similar between the groups. The relative wall thickness (p = 0.002) and LV mass index (p = 0.071) were more pronounced in the moderate aortic stenosis compared to the mild group. Both fibulin-1 (p = 0.045) and NT-proBNP (p = 0.018) levels were higher in the moderate aortic stenosis group, whereas suPAR levels were similar (p = 0.44). After one year, this difference in levels of fibulin-1 (71.5 vs. 67.3 µg/mL), NT-proBNP (179.5 vs. 138.9 ng/L) and suPAR (3.7 vs. 3.6 ng/mL) prevailed (all, p<0.05). Whereas, after four years the differences in fibulin-1 (71.3 vs. 67.7 µg/mL), NT-proBNP (198.8 vs. 159.7 ng/L) and suPAR (3.55 vs. 3.56 ng/mL) were not significantly any more.

**Table 1:** Clinical and descriptive characteristics of mild vs. moderate AS patients.

	AVAI ≥0.58 cm^2^/m^2^ n = 180	AVAI <0.58 cm^2^/m^2^ n = 194	p-value
Age, yrs.	66.1±9.9	66.3±9.3	0.81
Gender (male/female), n	109/73	130/72	0.37
Body surface area, m^2^	1.88±0.21	1.94±0.19	0.002
Treatment vs. Placebo,%	53.3/46.7	48.02/51.98	0.30
*Biochemical analyses*			
Fibulin-1, µg/mL	67.5±19.7	71.2±16.7	0.045
NT-proBNP, pg/mL	138.9 (30.0–745.8)	179.5 (27.2–1414.6)	0.018
suPAR, ng/mL	3.6±1.11	3.7±1.24	0.44
C-reactive protein, mg/L	0.21 (0.03–1.27)	0.19 (0.03–0.96)	0.37
Serum creatinine, mg/dL	95.2±15.7	97.2±15.5	0.20
Aspartate aminotransferase, U/L	17.7±6.7	16.8±4.02	0.12
γ-glutamyl transferase, U/L	24.9±25.5	23.7±20.9	0.61
*Cardiovascular measurements*			
Systolic blood pressure, mm Hg	143.2±19.3	144.9±20.0	0.41
Diastolic blood pressure, mm Hg	81.5±9.3	82.2±9.4	0.46
Ejection fraction,%	65.5±8.7	64.7±8.4	0.34
Fractional shortening,%	36.7±6.7	35.9±6.3	0.28
Left ventricular mass index, g/m^2^	95.5±26.8	100.6±28.6	0.071
Aortic valve area index, cm^2^/m^2^	0.75±0.142	0.47±0.074	<0.0001
Relative wall thickness	0.32±0.07	0.34±0.08	0.002

Values are arithmetic mean ± SD, geometric mean (5th and 95th percentiles) or the number of participants indicated as percentage. Left ventricular mass and Aortic valve area were indexed by Body surface area.

In unadjusted analysis, we explored fibulin-1′s relationship with echocardiography measures in the total group (Table 2). Fibulin-1 correlated inversely with AVAI at baseline (p = 0.001), 1 year follow-up (borderline p = 0.062) and 4 year follow-up (p<0.001), but no significant trend was observed with LV mass index, aortic regurgitation, LV ejection fraction and mid-wall shortening (only baseline data available) at any time point. Change in aortic stenosis progression, bicuspid/tricuspid aortic valve myocardial hypertrophy and LV function were not related to baseline fibulin-1 or changes in fibulin-1 (all p>0.099; data not shown). Notably, bicuspid aortic valve (n = 21) did not interact with the association of fibulin-1 with NT-proBNP (β = −0.147; p = 0.52) compared to patients with tricuspid valve (β = 0.133; p = 0.029). Similarly, bicuspid aortic valve did not interact with the association of fibulin-1 with suPAR at baseline (β = −0.005; p = 0.99), 1 year (β = 0.323; p = 0.396) and 4 years (β = 0.382; p = 0.557) (interaction data not shown).

**Table 2:** Pearson correlation coefficients of fibulin-1 with aortic and cardiac measures and relative changes in cardiac measurements.

	Fibulin-1 (log µg/mL)			
	Baseline	1 Year follow-up	4 year follow-up	% change in measurement
Aortic valve area index (log cm^2^/m^2^)	r = −0.173; p = 0.001	r = −0.103; p = 0.062	r = −0.218; p<0.0001	−4.05
Left ventricular mass index (log g/m^2^)	r = −0.078; p = 0.14	r = −0.071; p = 0.19	r = −0.006; p = 0.91	21.99
Left ventricular ejection fraction (%)	r = −0.028; p = 0.61	r = −0.148; p = 0.78	r = −0.091; p = 0.087	1.62
Left ventricular outflow maximum gradient (mm Hg)	r = −0.129; p = 0.001	r = −0.051; p = 0.35	r = −0.143; p = 0.007	0.339
Transaortic time velocity (msec)	r = 0.101; p = 0.045	r = 0.079; p = 0.15	r = 0.082; p = 0.12	28.93
Stress-corrected midwall shortening (%)	r = 0.010; p = 0.85	–	–	–

**Figure 1:**
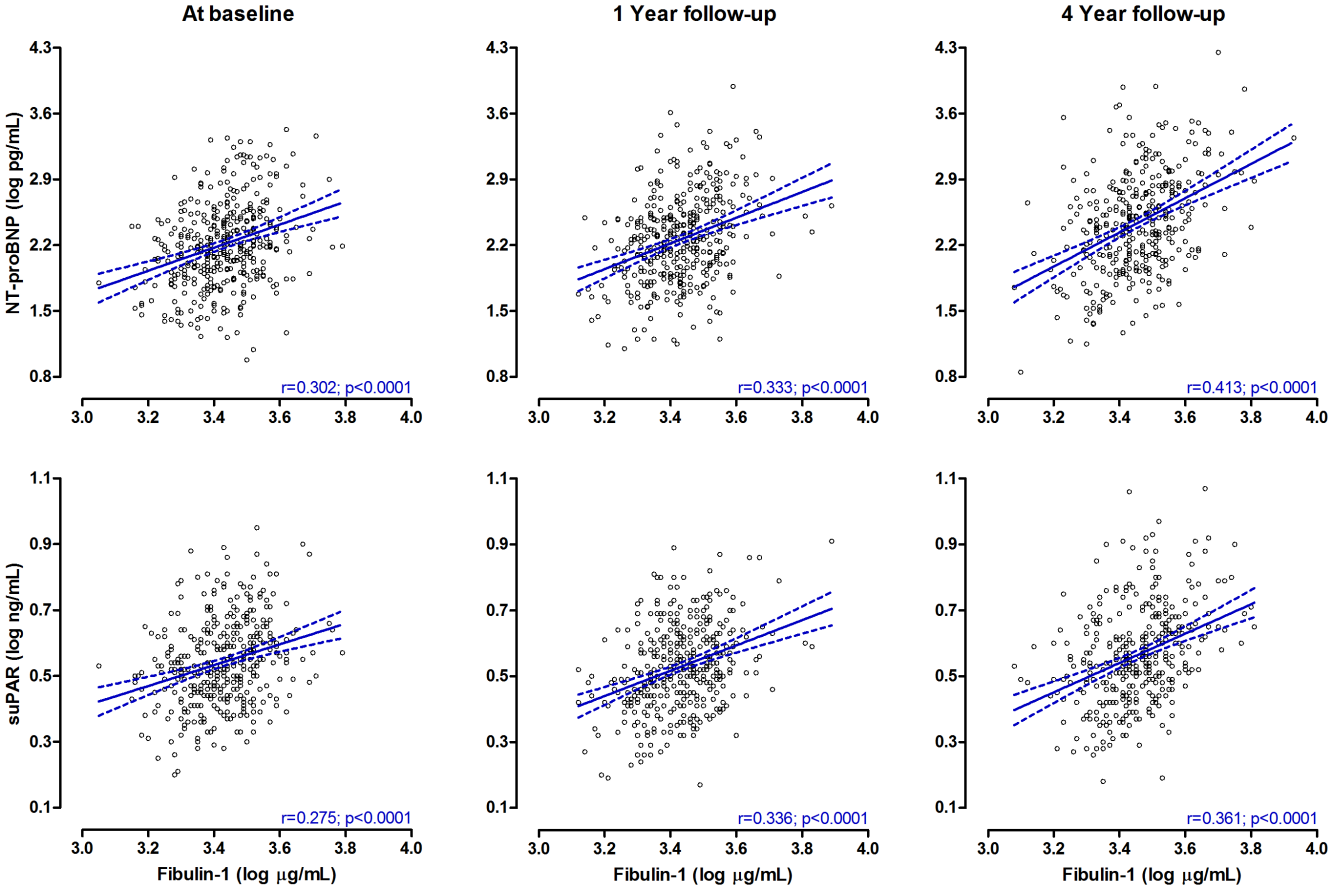
Unadjusted correlations of fibulin-1 with NT-proBNP and suPAR in patients with aortic stenosis during 4 years of follow-up.

**Figure 2:**
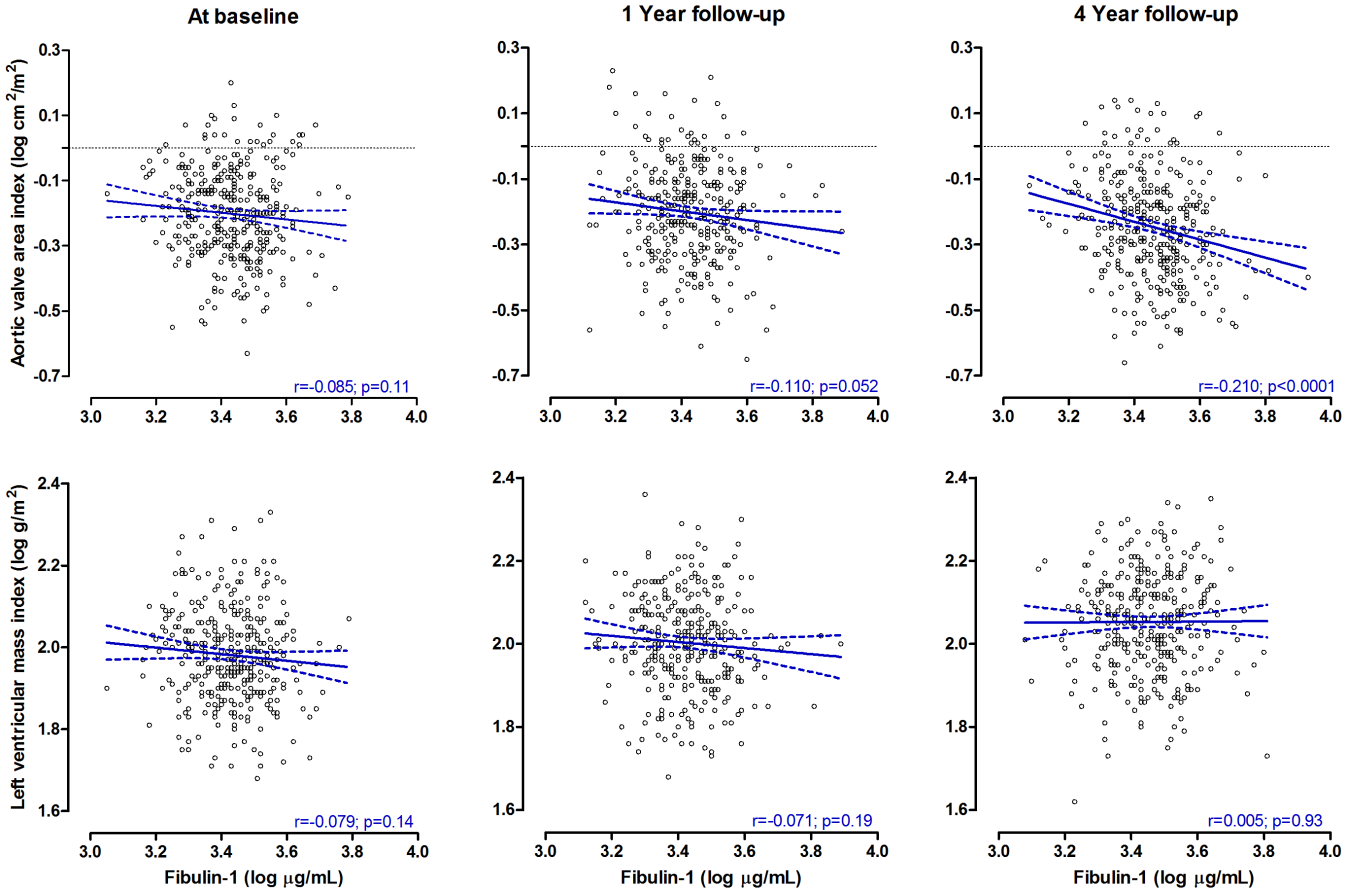
Unadjusted correlations of fibulin-1 with AVAI and LV mass index in patients with aortic stenosis during 4 years of follow-up.

Neither gender, AVAI category nor treatment allocation interacted significantly on the association of fibulin-1 with NT-proBNP and suPAR, respectively. In Figure 1, fibulin-1′s relationship with NT-proBNP and suPAR were plotted for the total population at baseline, after 1 year and 4 years of treatment. The regression coefficients of fibulin-1 in relation to NT-proBNP and suPAR, strengthened at each follow-up interval (all, p<0.001). Fibulin-1 showed a significant inverse relationship with AVAI at 4 years follow-up only (r = -0.21; p<0.001), but a borderline trend was observed between fibulin-1 and AVAI after 1 year follow-up (p = 0.052, Figure 2).

Although Simvastatin and Ezetimibe treatment did not influence the correlation of fibulin-1 with NT-proBNP and suPAR, we tested the partially adjusted (age, body mass index and serum creatinine) correlations of fibulin-1 with NT-proBNP, suPAR and echocardiography measures stratified by treatment allocation (Table S1 in File S1). Independently of age, body mass index and creatinine, fibulin-1 significantly correlated with NT-proBNP (p = 0.001), suPAR (p = 0.007) and AVAI (p = 0.007) at baseline. With the exception of no correlation between fibulin-1 and AVAI in the treated group (p = 0.57), fibulin-1 significantly correlated with NT-proBNP and suPAR in both placebo vs. treated group (all. p<0.01), and also with AVAI in the placebo group (p = 0.009). The same trend was observed after 4 years follow-up, but with stronger correlations and also a borderline tendency of fibulin-1 with AVAI in the treated group. Partially adjusted correlations of fibulin-1 with other echocardiography measures are also listed in Table 2s, although no significant relationship existed. ANCOVA was performed to test the relationship of fibulin-1 with NT-proBNP and suPAR at 1 and 4 years follow-up independent of age, treatment allocation and baseline fibulin-1 levels. At 1 year follow-up, fibulin-1 correlated significantly with NT-proBNP (r = 0.218; p<0.001) and suPAR (r = 0.132; p = 0.009) and even stronger correlations were observed at 4 years follow-up (NT-proBNP (r = 0.328; p<0.001) and suPAR (r = 0.166; p = 0.002)).

A multiple regression analysis was performed to show the associations of fibulin-1 with NT-proBNP and suPAR independently of age, gender, serum aspartate aminotransferase, serum creatinine and treatment allocation (Table 3). At baseline fibulin-1 associated inversely with AVAI and aspartate aminotransferase, but not with NT-proBNP and suPAR. After 1 year follow-up period the association of fibulin-1 with NT-proBNP (β = 0.16; p = 0.005) and suPAR (β = 0.16; p = 0.006) became evident and the association with aspartate aminotransferase remained (β = 0.15; p = 0.007), but the significant association with AVAI disappeared (p = 0.11). After 4 years the same trend was observed, but with only a borderline association between fibulin-1 and aspartate aminotransferase (p = 0.067). In exploratory analysis we performed the same multivariate analysis after dividing the group into higher and lower AVAI category (Tables S2 and S3 in File S1). In the mild AS group (Table S2 in File S1) fibulin-1 associated significantly with NT-proBNP at 4 years follow-up (p = 0.007) and with suPAR at both 1 year (p = 0.040) and 4 years (0.036) of treatment. In the moderate AS group (Table S3 in File S1) fibulin-1 associated strongly with NT-proBNP at baseline (p = 0.001), 1 year (p = 0.003) and 4 years (p = 0.004) of treatment and with suPAR at both 1 year (p = 0.003) and 4 years (p<0.001) follow-up.

**Table 3:** Multiple regression analysis of fibulin-1, NT-proBNP and suPAR in patients with AS at baseline and after one and four years of treatment.

	Fibulin-1, µg/mL							
	BASELINE		1 YEAR FOLLOW-UP			4 YEARS FOLLOW-UP		
*R^2^*	0.20			0.24			0.28	
*Adjusted R^2^*	0.19			0.22			0.27	
	**Standard β**	**p value**		**Standard β**	**p value**		**Standard β**	**p value**
NT-proBNP, pg/mL	0.097	0.079		0.160	0.005		0.224	<0.0001
suPAR, ng/mL	0.053	0.34		0.163	0.006		0.129	0.026
Aortic valve area index, cm^2^/m^2^	−0.143	0.005		−0.084	0.11		−0.063	0.22
Age, years	0.254	<0.0001		0.166	0.005		0.189	0.001
Gender	0.146	0.011		0.192	0.001		0.187	0.001
Aspartate aminotransferase, U/L	0.161	0.002		0.149	0.007		0.091	0.067
Serum creatinine	0.064	0.26		0.058	0.32		0.098	0.080
Treatment	–	–		−0.026	0.62		−0.010	0.84

Multivariate analysis was performed independently of age, gender, aspartate aminotransferase levels and treatment allocation.

Finally, we performed the same regression but using the MDRD Study equation for eGFR instead of serum creatinine only. We observed a significant association between fibulin-1 and NT-proBNP (β = 0.26; p<0.001) at the first visit after baseline, and with both NT-proBNP (β = 0.25; p<0.001) and AVAI after 4 years (β = −0.13; p = 0.029).

## Discussion

The current study has two new observations: higher fibulin-1 levels were associated with higher suPAR and NT-proBNP levels, especially in patients with lower AVAI; and this association persisted during 4 years of observation independent of age, gender, serum creatinine and treatment intervention. These observations suggest that the association at least partly was due to the increased afterload and inflammation associated with the stenotic aortic valve. The levels of fibulin-1 and NT-proBNP increased independently parallel to increasing severity of the aortic stenosis. The interplay of these biomarkers in aortic stenosis patients indicates the detrimental overload and subsequent adverse changes of the local extracellular matrix scaffold within the myocardium in relation to decreasing aortic valve area.

Although definite mechanisms on fibulin-1′s role in extracellular matrix remodelling, especially in clinical situations such as type 2 diabetes, atherosclerosis, cardiac ischemia and heart failure, remain to be elucidated, some evidence exists indicating its role in some of these conditions [8], [11]. The undertone of fibulin-1 seems to be driven by potential vascular dysfunction and subsequent extracellular matrix turnover within the cardiovascular tissue. A previous study reported the association between fibulin-1 and NT-proBNP in a healthy cohort of South African men, suggesting that high levels of plasma fibulin-1 along with ventricular secretion of NT-proBNP indicates early morphological changes within blood vessels and cardiac tissue [12]. This is in line with a study conducted earlier by Cangemi et al. that described the augmented concentrations of fibulin-1 in arterial walls and in plasma of type 2 diabetic patients, and may propose vascular extracellular matrix turnover as shown by their association between fibulin-1 and arteriosclerosis or arterial stiffness [11].

Our study population was clinically diagnosed with asymptomatic aortic stenosis, particularly chosen to evaluate fibulin-1′s role in fibrotic and sclerotic processes in relation to cardiac overload and subclinical atherosclerosis-related inflammation. Since this study only evaluated the relationship between biomarkers at baseline and after drug intervention we cannot infer causality. However, this study along with on-going investigations attempts to explain the potential pathway in which these biomarkers are linked. Aortic valve stenosis has similar characteristics with coronary artery disease which include adverse lipid build-up, inflammation and medial calcific lesions [1]. Hemodynamic strain also contributes to valvular thickening, further tightening the orifice of the aortic valve and causing a systolic shunt at the LV outflow [1], [22]. It has been shown that LV hypertrophy is related to myocardial sclerosis [23]. However, the link between fibulin-1 and NT-proBNP in our study was unrelated to aortic regurgitation and LV ejection fraction, but linked closely with the AVAI. The lack of association between fibulin-1 and echocardiography data may be due to unrecognised myocardial fibrosis in the asymptomatic and less severe nature of aortic stenosis of this cohort. This hypothesis that fibulin-1 reflects asymptomatic myocardial fibrosis that is not accompanied by LV hypertrophy and/or reduced ejection fraction, give rise to the possibility that fibulin-1 may add prognostic information to the current echocardiographic follow-up in these patients.

Fibulin-1 levels were augmented in our patients with more severe aortic stenosis and this confirms the observation previously described by Scholze et al [24] showing an association to LV systolic load. Since we also found a relationship between fibulin-1 and suPAR along with presented hemodynamic strain in this cohort, our study propose cross-linking between the plasminogen pathway and fibrotic processes within the extracellular matrix. Soluble uPAR has a distinct role in cell adhesion, proliferation and migration between cells in the arterial wall. These cells include endothelial cells, smooth muscle cells and macrophages in atherosclerotic progression [25]. In addition, plasma lipoproteins and molecular pathways involved in thrombosis, fibrinolysis, oxidation and inflammation are regulated by the urokinase-type plasminogen activator (uPA) and its receptor (uPAR) [26], [27]. Disproportion in this system leads to disorders in tissue structure and function [25]. Fibulin-1 coincides in the plasminogen system by means of its shared role in cell adhesion, especially from its site of secretion in arterial walls and its interaction with plasma fibrinogen and calcium to promote platelet adhesion [10]. The cross-linking of fibulin-1 with its local extracellular matrix proteins may explain its role within exposed sub-endothelial matrices following vascular disease. In a study earlier this year, NT-proBNP was found to be associated with suPAR, which suggested that hemodynamic strain on the heart is associated with low-grade inflammation [28]. This is due to either systemic inflammation or acute phase responses that contribute to extracellular matrix remodelling within cardiac tissue as a result of increased volume overload [29]. In addition, Eugen-Olsen et al. showed that suPAR associates with an increased risk of cardiovascular disease, cancer, type 2 diabetes and mortality [30]. The interaction between fibulin-1, NT-proBNP and suPAR is therefore warranted to explain the development of sclerotic and fibrotic disease states.

In studies with animals subjected to experimental hypercholesterolemia it was confirmed that elevated lipid accumulation causes both atherosclerosis and contribute to calcification in aortic valves [31]–[35]. In our study, patients were subjected to lipid lowering treatment or placebo and even with extreme lipid lowering drug interventions, the association of fibulin-1 with both a biomarker of cardiac strain and one of subclinical atherosclerotic inflammation remained after one and four years of treatment, taking into consideration a measure of renal function, gender, age and the liver enzyme aspartate aminotransferase. This result suggests that fibulin-1 may indicate valvular or myocardial fibrosis independent of treatment allocation. Although gender associated significantly in the regression model, it is worthy to mention that suPAR levels are generally higher in women compared to men and that the multiple linearity of this gender difference may reflect an interaction, although our interaction tests revealed no impact of gender. With fibulin-1 connected to both NT-proBNP and suPAR via different physiological pathways and in various disease states, more studies are encouraged to investigate and define the concise mechanism of the relationship between these biomarkers.

### Limitations

This study was conducted from a sample selected from the larger SEAS study, since the relevant biomarkers were not available in the entire study population. The study sample was limited to patients with aortic stenosis as defined by aortic valve peak velocities of ≥2.5 and ≤4.0 m/sec by echocardiography. MRI data on aortic stenosis or myocardial fibrosis was not acquired in this study. Other methods for quantifying fibrosis are known, for example late gadolinium enhancement or cardiac magnetic resonance contrast-enhanced T1-mapping. These methods were not available in this study and therefore fibulin-1 may aid as surrogate marker or add to depicting potential fibrosis onset in cardiovascular tissue. Comparing the use of fibulin-1 with known methods is therefore encouraged.

In conclusion, elevated levels of plasma fibulin-1 were associated with aortic stenosis, elevated LV hemodynamic load as indicated by elevated levels of NT-proBNP and soluble uPAR supporting a possible link in cardiovascular extracellular sclerotic processes. This prospective study suggests that fibulin-1 and soluble uPAR are involved in myocardial extracellular matrix turnover as a result of elevated LV overload in patients with aortic stenosis.

## Supporting Information

File S1 **Supporting tables. Table S1.** Partial correlation coefficients of fibulin-1 with NT-proBNP, suPAR and aortic/cardiac measures at baseline and follow-up in patients with aortic stenosis stratified by treatment allocation. **Table S2.** Multiple regression analysis of fibulin-1 and NT-proBNP in patients with mild AS at baseline and after one and four years of treatment. **Table S3.** Multiple regression analysis of fibulin-1 and NT-proBNP in patients with moderate AS at baseline and also after one and four years of treatment.
(DOCX)
