# Supplementary material for: Extracellular Matrix Biomarker, Fibulin-1, Is Closely Related to NT-proBNP and Soluble Urokinase Plasminogen Activator Receptor in Patients with Aortic Valve Stenosis (The SEAS Study)
Source: PLoS One. 2014 Jul 11;9(7):e101522. doi: 10.1371/journal.pone.0101522 (PMC4094491; doi:10.1371/journal.pone.0101522)
Supplement: File S1 — Supporting tables. Table S1. Partial correlation coefficients of fibulin-1 with NT-proBNP, suPAR and aortic/cardiac measures at baseline and follow-up in patients with aortic stenosis stratified by treatment allocation. Table S2. Multiple regression analysis of fibulin-1 and NT-proBNP in patients with mild AS at baseline and after one and four years of treatment. Table S3. Multiple regression analysis of fibulin-1 and NT-proBNP in patients with moderate AS at baseline and also after one and four years of treatment. (DOCX) [file pone.0101522.s001.docx]

|  | **Fibulin-1, log μg/mL** | | | | |
| --- | --- | --- | --- | --- | --- |
|  | **Baseline** | **1 Year follow-up** | | **4 year follow-up** | |
|  |  | **Placebo** | **Treated** | **Placebo** | **Treated** |
| NT-proBNP, pg/mL | r=0.175; p=0.001 | r=0.222; p=0.002 | r=0.238; p=0.001 | r=0.354; p<0.0001 | r=0.262; p<0.0001 |
| suPAR, ng/mL | r=0.135; p=0.007 | r=0.302; p<0.0001 | r=0.204; p=0.004 | r=0.317; p<0.0001 | r=0.225; p=0.002 |
| Aortic valve area index, log cm^2^/m^2^ | r=–0.143; p=0.007 | r=–0.208; p=0.009 | r=–0.044; p=0.57 | r=–0.211; p=0.006 | r=–0.127; p=0.085 |
| Left ventricular mass index, log g/m^2^ | r=–0.072; p=0.18 | r=–0.055; p=0.48 | r=–0.044; p=0.57 | r=0.005; p=0.94 | r=–0.001; p=0.99 |
| Aortic regurgitation, cm | r=–0.023; p=0.68 | r=0.004; p=0.96 | r=–0.078; p=0.31 | r=–0.117; p=0.13 | r=–0.056; p=0.45 |
| Ejection fraction, % | r=–0.015; p=0.78 | r=–0.032; p=0.69 | r=–0.057; p=0.47 | r=–0.081; p=0.29 | r=–0.021; p=0.77 |

**Table S1.** Partial correlation coefficients of fibulin-1 with NT-proBNP, suPAR and aortic/cardiac measures at baseline and follow-up in patients with aortic stenosis stratified by treatment allocation

Adjustments applied for: age, body mass index and serum creatinine.

**Table S2.** Multiple regression analysis of fibulin-1 and NT-proBNP in patients with mild AS at baseline and after one and four years of treatment

|  | **Fibulin-1, μg/mL** | | | | | | | |
| --- | --- | --- | --- | --- | --- | --- | --- | --- |
|  | **BASELINE** | | **1 YEAR FOLLOW-UP** | | | **4 YEARS FOLLOW-UP** | | |
| *R^2^* | 0.21 | | | 0.25 | | | 0.32 | |
| *Adjusted R^2^* | 0.19 | | | 0.22 | | | 0.29 | |
|  | **Standard β** | **p value** | | **Standard β** | **p value** | | **Standard β** | **p value** |
| NT-proBNP, pg/mL | 0.032 | 0.68 | | 0.109 | 0.14 | | 0.203 | 0.007 |
| suPAR, ng/mL | 0.076 | 0.31 | | 0.153 | 0.040 | | 0.158 | 0.036 |
| Age, years | 0.358 | <0.0001 | | 0.308 | <0.0001 | | 0.304 | <0.0001 |
| Gender | 0.194 | 0.092 | | 0.130 | 0.061 | | 0.160 | 0.020 |
| Aspartate aminotransferase, U/L | 0.132 | 0.058 | | 0.115 | 0.12 | | 0.067 | 0.33 |
| Treatment | – | – | | 0.063 | 0.37 | | –0.002 | 0.98 |

Multivariate analysis was performed independently of age, gender, aspartate aminotransferase levels and treatment allocation.

**Table S3.** Multiple regression analysis of fibulin-1 and NT-proBNP in patients with moderate AS at baseline and also after one and four years of treatment

|  | **Fibulin-1, μg/mL** | | | | | | | |
| --- | --- | --- | --- | --- | --- | --- | --- | --- |
|  | **BASELINE** | | **1 YEAR FOLLOW-UP** | | | **4 YEARS FOLLOW-UP** | | |
| *R^2^* | 0.17 | | | 0.26 | | | 0.29 | |
| *Adjusted R^2^* | 0.15 | | | 0.24 | | | 0.26 | |
|  | **Standard β** | **p value** | | **Standard β** | **p value** | | **Standard β** | **p value** |
| NT-proBNP, pg/mL | 0.250 | 0.001 | | 0.213 | 0.003 | | 0.223 | 0.004 |
| suPAR, ng/mL | 0.085 | 0.26 | | 0.228 | 0.003 | | 0.213 | 0.006 |
| Age, years | 0.117 | 0.142 | | 0.037 | 0.63 | | 0.124 | 0.12 |
| Gender | 0.178 | 0.014 | | 0.218 | 0.002 | | 0.214 | 0.002 |
| Aspartate aminotransferase, U/L | 0.168 | 0.022 | | 0.228 | 0.001 | | 0.194 | 0.006 |
| Treatment | – | – | | 0.054 | 0.41 | | 0.013 | 0.85 |

Multivariate analysis was performed independently of age, gender, aspartate aminotransferase levels and treatment allocation.
